# C-Reactive Protein Is a Poor Marker of Baseline Inflammation in Prostate Cancer and Response to Radiotherapy or Androgen Ablation

**DOI:** 10.7759/cureus.19639

**Published:** 2021-11-16

**Authors:** Garrett L Jensen, Jason Naziri, Kendall P Hammonds, Sameer G Jhavar, Gregory Swanson

**Affiliations:** 1 Radiation Oncology, Baylor Scott & White Medical Center - Temple, Temple, USA; 2 Radiation Oncology, Baylor Scott & White Health, Temple, USA; 3 Biostatistics, Baylor Scott & White Health, Temple, USA

**Keywords:** treatment response, inflammatory markers, prostate cancer, c-reactive protein, acute phase reactant, crp levels

## Abstract

Introduction

C-reactive protein (CRP) is an acute-phase reactant used as a general marker for inflammation. Isolated levels have been associated with prostate cancer development, prostate-specific antigen (PSA), Gleason score, and treatment response. We seek to establish whether CRP levels reflect inflammation caused by prostate cancer by comparing levels at various points of time before, during, and after therapy.

Materials and methods

A total of 209 patients had a complete blood count (CBC), PSA, and CRP taken at up to four different time points. Labs were performed up to one week prior to androgen ablation via leuprolide injection (pre-AA), up to one week prior to radiotherapy (RT) (pre-RT), within one week of RT completion (post-RT), and three months following RT completion (FU [follow-up]).

Results

Significant relationships were found between CRP and WBC pre-AA (p-value=0.0050), pre-RT (p-value=0.0170), and post-RT (p-value=0.0113), but not at FU (p=.096). CRP had no significant relationship with PSA or lymphocytes at any time points. PSA was significantly affected by androgen ablation but lymphocytes, WBCs, and CRP were not. No CRP levels were associated with risk groups or FU-PSA. Lymphatic radiation fields significantly decreased WBCs and lymphocytes but not CRP.

PSA, WBC, and lymphocytes all significantly decreased from pre-RT to post-RT, followed by a significant recovery. CRP did not significantly change during any of these periods and was not significantly related to changes in PSA, WBCs, or lymphocytes.

Conclusion

CRP is not a sensitive marker of the acute inflammatory effects of non-metastatic prostate cancer and treatment response with androgen ablation or radiation therapy.

## Introduction

C-reactive protein (CRP) is a pentameric acute-phase protein from the liver that is found in blood plasma and increases following interleukin-6 secretion by macrophages and T cells. It rises rapidly (dramatically, peaking within 24 hours in the case of other acute inflammatory reactions, including that induced by radiation exposure) [1] and has a half-life of 19 hours. Ostensibly, the greater the inflammation, the higher the CRP [2].

Historically, it was thought to be helpful in monitoring bacterial infections and their resolution [3]. It has also been used as a marker in recognized chronic inflammatory diseases such as rheumatoid and juvenile arthritis [1]. More recently it has been found to be prognostic for recurrent cardiovascular events which is thought to have a component of chronic inflammation [4,5].

Given the association between the immune system and the development and progression of cancer, it is not surprising that there would be interest as to whether CRP, as a measure of immune response, could help determine cancer prognosis. If CRP reflects the host inflammatory state, it may be a marker related to promotion and initiation or progression of carcinogenesis and/or mediate resistance to therapy.

The available literature on CRP in cancer largely focuses on pre-therapeutic levels as a prognostic marker for cancer development and treatment response [6,7]. The results of these papers are severely hampered by patient heterogeneity or the use of CRP measurements at a single, often variable time point [3,8-11]. Unfortunately, there are no comprehensive studies looking at CRP and its changes at different stages of cancer treatment, unlike the situation with infection where it improves with treatment [12]. This has made it difficult to conclude the true relationship between CRP as an accurate marker of cancer inflammation or treatment response [13]. Does killing the cancer or radiotherapy (RT)-induced inflammation change the CRP level? If the CRP does not change, is this because these factors offset each other? With longer follow-up, when the RT inflammation has resolved, is CRP more reflective of the cancer status?

Prostate cancer has been a focal point of many publications investigating serum CRP as a potentially important biomarker for nonmetastatic prostate cancer [14]. One study suggested that elevated CRP levels are prognostic of poor outcomes with radiotherapy in particular [15]. It is an affordable, readily available assay. Findings regarding an association between CRP levels and prostate cancer risk have been mixed [3,11,16-23]. Studies have also found some inconsistent relationships between CRP and components of higher-risk disease such as Gleason score, metastases, and prostate-specific antigen (PSA) [24-29]. This plethora of studies has resulted in several meta-analyses [10,30,31] and, particularly intriguing, a pooled analysis showing that CRP levels were associated with overall survival (OS) [32]. As with other cancer sites, these results can be unconvincing based on the timing and methodology of CRP level collection. Stronger evidence has accumulated that CRP can be a prognostic marker in the metastatic setting, which is associated with greater levels of inflammation [33-41].

We seek to establish whether CRP levels reflect inflammation caused by non-metastatic prostate cancer before and in response to RT with or without prior androgen ablation (AA).

## Materials and methods

With approval from the Baylor Scott & White Research Institute (IRB number 019-511), this study included 209 patients who had a complete blood count (CBC), PSA, and CRP taken at up to four different time points. Labs were performed prior to AA in castration-naive patients via leuprolide injection (pre-AA), within one week prior to starting RT (pre-RT), within one week of RT completion (post-RT), and three months following RT completion (FU). Prior to RT, 33% of patients had received prior prostatectomy and 51% had received AA. Radiation fields included pelvic lymphatics in 82% of patients.

Comparisons were made between WBC, lymphocytes, PSA, and CRP at identical and different time points as continuous variables or split into halves/quartiles by CRP levels. Statistical analysis involving PSA was done for the entire cohort and separately by the history of prostatectomy.

A linear regression assessed the ability of PSA to predict CRP at the same time point. CRP was log-transformed to meet model assumptions. PSA was also analyzed by median CRP of upper and lower halves at the same time points using a Wilcoxon rank-sum test with a Bonferroni adjusted p-value. PSA was then measured by CRP split into quartiles at the same time points with analysis via Kruskal-Wallis test, also using a Bonferroni adjusted p-value. These three statistical tests were repeated to assess the ability of CRP to predict WBC at the same time points, and the relationship between pre-AA, pre-RT, and post-RT CRP with the FU PSA.

An ordinal logistic regression was performed to assess the relationship between pre-AA and pre-RT CRP and Gleason score.

A Wilcoxon rank-sum test was performed to assess the difference in post-RT and FU PSA, CRP, lymphocytes, or WBCs by whether lymph nodes were irradiated. This test was also applied to assess the difference in pre-RT PSA, CRP, WBC, and lymph nodes by whether androgen ablation was employed prior to radiation.

A Wilcoxon signed-rank test was performed to assess if changes over time were significant. This included analyzing changes in pre-AA PSA, CRP, WBC, or lymphocytes to pre-RT levels either throughout the cohort or by whether AA was employed prior to radiation. To look at radiation’s effect on immunity this test was used for changes in PSA, CRP, WBC, and lymphocytes from pre-RT to post-RT and pre-RT to FU. A Spearman Rank Correlation test was performed to further the relationship between the change in CRP and the changes in PSA, WBC, and lymphocytes during these timepoints. Statistical significance is set to p<0.05.

## Results

For patient characteristics, see Table 1. There were several major variables. There were patients with intact prostate (n=139) and those with post-prostatectomy (n=70). All but nine patients received radiation, to the prostate or prostate fossa, with some receiving radiation to the pelvic lymphatics (n=162). Finally, some received androgen ablation prior to radiation or observation (n=106). We took each of these variables into consideration. Neither pre-AA CRP nor pre-RT CRP was correlated with Gleason score (p=0.43 and 0.22, respectively).

**Table 1:** Patient Characteristics EBRT: External beam radiotherapy; BT: Brachytherapy; AA: Androgen ablation; RT: Radiotherapy; PSA: Prostate-specific antigen.

Patient Characteristics		
		n (%)
Radiation	None	9 (4.3)
	EBRT	186 (89)
	EBRT + BT	14 (6.7)
Race	Caucasian	133 (63.6)
	African American	52 (24.9)
	Hispanic	18 (8.6)
	Other	6 (3)
Gleason (clinical)	6	29 (14.4)
	7 (3+4)	59 (29.4)
	7 (4+3)	56 (27.9)
	8	34 (16.9)
	9	22 (11.0)
	10	1 (0.5)
Radical Prostatectomy	None	139 (66.5)
	Yes, with salvage RT	51 (24.4)
	Yes, with adjuvant RT	19 (9.1)
AA prior to RT/Observation	Yes	106 (50.7)
	No	103 (49.3)
Lymph nodes treated	No	38 (19.0)
	Yes	162 (81.0)
PSA at diagnosis (ng/mL), median (range)		8.8 (1.2-278)

Median values and ranges of PSA, CRP, WBC, and lymphocytes are reported in Table 2. Inherent to the patient populations (intact prostate versus post-prostatectomy) there was a significant difference between PSA at all time points (p<0.0001) between the two groups. Since PSA correlates with cancer volume, this is indicative of the significantly different cancer volumes between the two groups. It is therefore informative that these same populations had no significant difference in CRP at any time point. Similarly, globally there was no correlation between PSA and CRP at baseline or any other time point (Table 3). CRP levels at any time point were not predictive of subsequent follow-up PSA. One would expect that as the cancer regresses (as measured by a declining PSA), so would the inflammatory response, and thus the CRP, which was not seen. This was true not only after the radiation, but also for those that received initial androgen ablation where only PSA significantly changed from pre-AA to pre-RT in both the no-surgery and surgery subsets (p<0.0001 and 0.0156, respectively). Levels of CRP, WBCs, and lymphocytes did not significantly change, indicating no immediate effect of androgen deprivation (Table 4). These would indicate no effect of a regressing cancer on those parameters.

**Table 2:** Values at Each Time Point ^a^ Significant difference between surgery and no surgery groups (P-value <0.0001) ^b^ No significant difference between surgery and no surgery groups (P-values of 1, 0.96, 0.74, and 0.36, respectively) ^c^ Significant or nearly significant difference between surgery and no surgery groups (P-value=0.0141, 0.0585, 0.0013, and 0.023, respectively) AA: Androgen ablation; RT: Radiotherapy; PSA: Prostate-specific antigen; CRP: C-reactive protein; WBC: White blood cells.

		Prior to AA		Prior to RT		Final Week of RT		3 Months Post-RT	
Labs	Prostatectomy History	n	Median (range)	n	Median (range)	n	Median (range)	n	Median (range)
PSA (ng/mL)	all	68	9.1 (0-155.5)*	187	1 (0.0-39.9)^a^	49	0.0 (0.0-11.4)^a^	190	0.1 (0.0-14.9)^a^
	no	54	10.0 (3.7-155.5)	127	2.8 (0.0-39.9)	23	1.5 (0.0-11.4)	124	0.6 (0.0-14.9)
	yes	14	0.6 (0.0-23.3)	60	0.2 (0.0-3.1)	26	0.0 (0.0-1.7)	66	0.0 (0.0-3.3)
CRP (μg/mL)	all	46	1.4 (0.2-71.5)^b^	172	2.6 (0.0-35.2)^b^	177	2.2 (0.0-76.3)^b^	176	2.3 (0.0-41.1)^b^
	no	39	1.5 (0.2-71.5)	110	2.5 (0.0-30.6)	113	2.2 (0.0-57.7)	114	2.4 (0.0-41.1)
	yes	7	1.2 (0.2-27.6)	62	2.9 (0.0-35.2)	64	2.1 (0.0-76.3)	62	2.1 (0.0-15.2)
WBC (10^3^ cells/μL)	all	83	6.9 (3.8-14.8)^b^	200	6.5 (3-14.8)^b^	191	4.7 (2.2-13.0)^c^	190	5.0 (2.1-14.7)^c^
	no	66	6.7 (4.0-8.8)	133	6.5 (3.0-14.8)	123	4.9 (2.4-13.0)	124	5.2 (2.1-14.7)
	yes	17	7.0 (3.8-14.8)	67	6.4 (4.0-11.0)	68	4.6 (2.2-11.1)	66	4.8 (2.2-8.1)
Lymphocytes (10^3^ cells/μL)	all	79	1.7 (0.8-4.0)^b^	198	1.9 (0.6-5.0)^b^	191	0.67 (0.2-3.3)^c^	187	0.9 (0.3-3.6)^c^
	no	62	1.8 (0.8-4.0)	131	1.9 (0.6-5.0)	123	0.7 (0.2-3.3)	122	1.0 (0.3-3.6)
	yes	17	1.6 (0.8-2.7)	67	1.9 (0.8-4.9)	68	0.6 (0.2-1.3)	65	0.8 (0.3-1.9)

**Table 3:** Association of CRP with PSA, WBC and Lymphocytes at Each Time Point ^a^ (10^3^ cells/μL) ^b^ (μg/mL) AA: Androgen ablation; RT: Radiotherapy; PSA: Prostate-specific antigen; CRP: C-reactive protein; FU: Follow-up; WBC: White blood cells.

	% increase of PSA per 1-unit CRP increase	p-value	% change of WBC per 1-unit^a^ CRP increase	p-value	% increase of lymphocytes per 1-unit^b^ CRP increase	p-value
pre-AA	0.4%	0.7525	0.8%	0.0028	0.46%	0.202
pre-RT	0.9%	0.4994	0.8%	0.0316	0.07%	0.897
post-RT	2.8%	0.0871	0.5%	0.0113	0.55%	0.1605
3-month FU	0.7%	0.2772	0.7%	0.0658	0.3%	0.5683

**Table 4:** The Effects of Androgen Ablation ^a^ Significant difference by androgen ablation ^b^ Significant change through time ^+^ No significant difference by surgical status for CRP, WBC, or Lymphocytes RT: Radiotherapy; PSA: Prostate-specific antigen; CRP: C-reactive protein; WBC: White blood cells; AA: Androgen ablation.

		With Androgen Ablation (n=106)	Without Androgen Ablation (n=103)	
		Median (range)	Median (range)	p-value
pre-RT PSA (all) +		0.9 (0-30.2)	2.85 (0.0-39.9)	0.0072^a^
	no surgery	1.0 (0.0-30.2)	7.4 (0.4-39.9)	<0.0001^a^
	surgery	0.0 (0.0-3.1)	0.2 (0.0-2.0)	0.7116
pre-RT CRP		2.35 (0-35.2)	2.85 (0.2-15.7)	0.7593
pre-RT WBC		6.3 (3-14.8)	6.55 (3.9-12.1)	0.5899
pre-RT Lymphocytes		1.82 (0.6-5.0)	1.94 (0.8-5.0)	0.3101
δpre-AA-->pre-RT PSA (all) +		-7.2 (-125.3-1.1)	N/A	<0.0001^b^
	no surgery	-8.1 (-125.3-1.1)		<0.0001^b^
	surgery	-0.75 (-20.7-0.1)		0.0156^b^
δpre-AA-->pre-RT CRP		-0.05 (-66.1-21.5)		0.646
δpre-AA-->pre-RT WBC		-0.1 (-5.8-4.4)		0.3598
δpre-AA-->pre-RT Lymphocytes		0.0 (-2.6-1.2)		0.9717

We further analyzed the relationship of the inflammatory parameters of WBC, CRP, and lymphocyte counts (Table 3). CRP had a significant correlation by linear regression with WBC levels pre-AA (p-value=0.0050), pre-RT (p-value=0.0170), and post-RT (p-value=0.0113), but not at FU (p=.096). CRP showed no significant correlation with lymphocyte levels.

**Figure 1:**
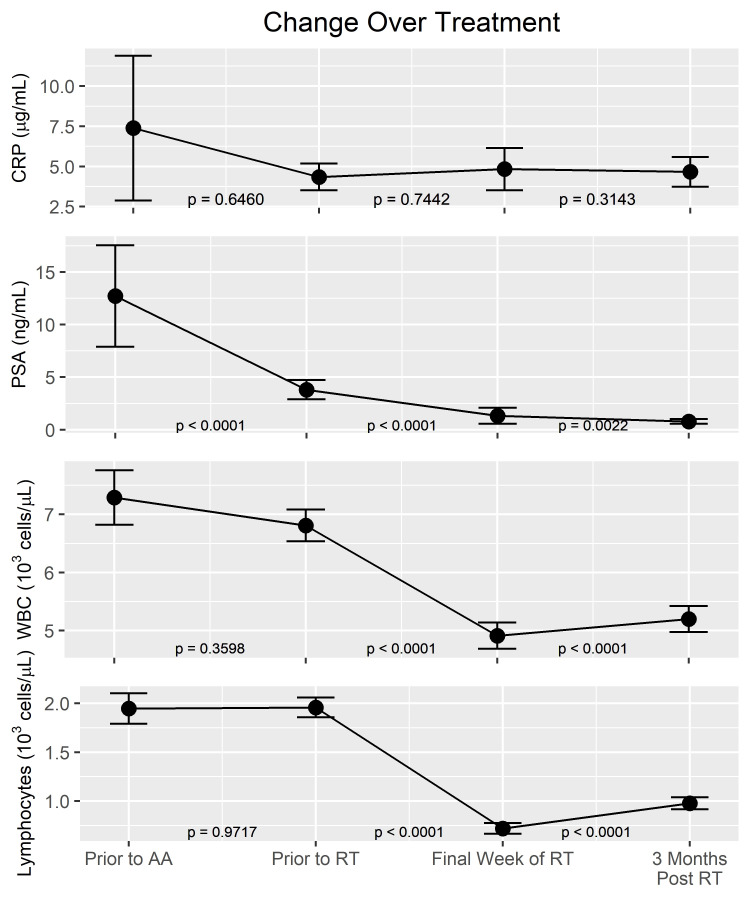
Change Over Time A comparison of changes over time of CRP, PSA, WBCs, and lymphocytes. P-values represent significant change over the specified interval with significance set to <0.05. Range of Y-axes for each lab value was based on maximum and minimum values of confidence intervals. CRP: C-reactive protein; PSA: Prostate-specific antigen; AA: Androgen ablation; RT: Radiotherapy.

PSA, WBCs, and lymphocytes significantly decreased from pre-RT to post-RT (p-values all <0.0001). This was the case with or without AA. PSA continued to decrease while WBCs and lymphocytes significantly recovered from post-RT to FU (p-value<0.0022, 0.0001, and 0.0001, respectively). Despite recovery, WBCs and lymphocytes from pre-RT to FU were still significantly decreased from baseline (p-value<0.0001, p-value<0.0001, respectively). Despite the significant ongoing changes in WBC and lymphocyte counts, CRP did not significantly change at any point (Figure 1). Specifically, the CRP did not significantly correlate with changes in WBCs or PSA. Singularly, a Spearman correlation was found between the change in lymphocytes and CRP from pre-RT to post-RT (p=0.0028). Also, in patients who received lymphatic radiation there was a significantly decreased post-RT lymphocytes (p-value<0.0001), FU-lymphocytes (p-value<0.0001), post-RT-WBCs (p-value=0.0004), and FU-WBCs (p-value=0.0141). CRP was not significantly different by lymphatic radiation post-RT (p-value=0.319) or at FU (p-value=0.712).

We did separate iterations of each aforementioned analyses with populations grouped into halves and quartiles by CRP to see if there might be a threshold of significance, but found no good evidence of a correlation with PSA, WBCs, or lymphocyte levels.

## Discussion

There is some evidence that elevated levels of CRP are associated with increased risk of prostate cancer development, PSA, Gleason score, and decreased response to chemotherapy and radiotherapy (RT). Usually, these are retrospective analyses of a single CRP level done before treatment without standard timing. Our data is the first presented in the literature to track CRP levels at different phases of treatment in any cancer. Prostate cancer was chosen for this study due to the existence of PSA as a rough marker for cancer activity, mass, and treatment response. We used this marker along with WBCs and lymphocytes to investigate the role of CRP in prostate cancer-based inflammation and the body’s inflammatory response.

CRP as a risk factor in cancer development

Investigations into CRP as a measure of risk for cancer development are part of what makes this marker intriguing. Prospective population studies and the literature overall have had mixed findings, but seem to support the ‘induction hypothesis’ that elevated baseline CRP is significantly correlated with a risk for overall cancer development [10,42,43]. Findings regarding specific cancer types are even more sporadic. Inconsistently positive results for breast and colorectal cancer could be a result of increased CRP levels due to an already present but undiscovered malignancy [3,11]. This is known as the ‘response hypothesis,’ where tumors induce an inflammatory microenvironment and a host response. The strongest statistical association appears to be with lung cancer, where elevated CRP is less likely to be due to secondary inflammation from an undiagnosed cancer due to short survival times [42].

Findings regarding the correlation between prostate cancer risk and CRP have also been mixed. In one large prospective study, CRP, fibrinogen, and leukocyte counts were all associated with colorectal, lung, and breast cancer but none were associated with prostate cancer [3]. Studies based on pre-diagnostic CRP levels have shown that other (BMI, IL-6 in healthy but not overweight men, leukocytes, metabolic syndrome components) or multiple etiologies of chronic inflammation correlate with increased prostate cancer risk, but often not CRP individually [20,21,26].

CRP and Gleason score

Relationships between baseline CRP, high-risk prostate cancer, and elevated PSA have been reported but not consistently replicated [24,44]. CRP polymorphisms and allelic variants have also failed to demonstrate an effect on prostate cancer risk or survival, though associations with higher-grade disease and PSA have been reported [17,18]. For example, one study showed no correlation of CRP with poor prognostic factors such as Gleason score or elevated PSA [15]. Another did show a correlation [9]. So, the correlation between prognostic factors and severity of the cancer as measured by CRP has remained uncertain. We did not find any correlation between pre-AA CRP or pre-RT CRP and clinical Gleason score in patients treated with definitive radiation.

CRP as a marker of acute cancer and treatment-based inflammation

CRP and PSA are most significantly tied to the extent of disease in the metastatic setting with some evidence for overall correlation [38,45]. Lehrer et al. found that CRP was not statistically different between men with localized prostate cancer and benign prostatic hyperplasia (BPH). However, patients with bone metastases had significantly higher CRP levels [27]. Several studies have looked at the CRP levels of patients with metastatic castration-resistant prostate cancer. In this setting, CRP appears to be prognostic for OS, progression-free survival (PFS), and PSA response to docetaxel [28,36,37,39]. CRP has also demonstrated a correlation with cancer-specific survival (CSS), and freedom from PSA failure in metastatic castration-resistant prostate cancer prior to abiraterone and enzalutamide [33]. CRP was built into the AFFINITY trial, which evaluated the clinical benefit of cabazitaxel alone or in combination with custirsen in metastatic castration‐resistant, docetaxel‐pretreated patients (NCT01578655) but results were not reported.

Unfortunately, without knowing whether there are changes in CRP, association with worse outcomes in these studies does not clarify whether CRP contributes to therapeutic resistance or is just a marker of disease and host factors. Less literature is available looking at CRP levels immediately before or after therapy in localized prostate cancer and none have reviewed CRP level changes based on therapy. Prior to radical prostatectomy, Schnoeller et al. found a significant relationship between CRP levels and Gleason score on univariate but not multivariate analysis without correlation to PSA, stage, lymph node positivity, or margin status [29]. Thurner et al. found that CRP levels >/= 8.6 prior to androgen deprivation therapy (ADT) or radiation were prognostic for CSS, OS, and disease-free survival (DFS) [15]. Hall et al. looked at the CRP levels of 206 patients within 30 days after they received radiotherapy and found that higher levels indicated a shorter Biochemical Failure Free survival (BFFS). On exploratory subgroup analysis, CRP levels also positively correlated with PSA in high and intermediate-risk cancer [9].

There is a distinct lack of data on CRP change before, during, and after treatment. We had the opportunity to evaluate each of these.

We looked at the association of PSA with CRP, WBCs, and lymphocytes prior to androgen ablation and prior to radiation treatment. We found no association between PSA and the markers, which suggests little direct correlation between prostate cancer volume and inflammation, at least in the non-metastatic setting. Neither was there any association between CRP and baseline or pre androgen ablation WBC or lymphocyte levels. In reports of the prognostic utility of CRP, cutoff levels of between 8-10 were used [9,43,46].

Next, we looked at the effect of androgen suppression in patients with pre-ADT PSA, CBC, and/or CRP levels and how things evolved three months after androgen deprivation, prior to radiation therapy. Androgen deprivation therapy clearly suppresses prostate cancer, and therefore should suppress the inflammation induced by that cancer. Androgens can enhance a T helper cell type 1 response [47] and peripheral Th1 and Th17 cells have been demonstrated to decrease after 24 months of ADT [48]. Other inflammatory cytokines with demonstrated reduction after ADT in prostate cancer patients include IL-1β, IL-2, tumor necrosis factor (TNF)-α, and interferon (IFN)-γ [49,50]. IL-6, which is linked with increased CRP expression, was demonstrated by Saylor et al. to be reduced by 12 months of ADT compared with non-ADT controls. Conversely, levels of IL-1β, IL-8, and SDF-1α were significantly higher [51]. Interestingly, in mice receiving radiation to the lungs, liver, and intestines, physical castration significantly enhanced the inflammatory response to radiation while flutamide did not. The underlying mechanism for this was thought to be due to increased NF-κB activity and subsequent elevated COX-2 with physical castration [52]. Unfortunately, these studies do not show whether these marker changes are a direct effect of ADT or are byproducts of cancer killing.

We found that CRP, WBC, and lymphocyte levels did not differ between patients who did and did not receive androgen ablation prior to radiation treatments. Unsurprisingly in our study, the PSA was significantly lower prior to radiation in those patients who did receive upfront androgen ablation. This expected PSA decline without appreciable change of CRP, lymphocytes, and WBCs appears to indicate that none of these factors accurately measure inflammatory changes from early prostate cancer suppression or eradication. Of course, there are other considerations. These changes may take longer, or there is a concomitant balancing increase in inflammation due to the cell killing. This finding that ablating tumor is poorly correlated with a decline in CRP has been demonstrated once before in a cohort of colorectal cancer patients [53].

Similarly, after the radiation treatment, there was an expected decline in leukocytes. The relationship between radiation therapy and cancer inflammatory response (immunity) is more complex. It is established that lymphocytes are probably the single most radiosensitive mammalian cell and radiation treatment significantly reduces lymphocyte counts. There were concerns raised decades ago that radiation-induced immune suppression would facilitate cancer recurrence or other immune compromise. Overall, that does not appear to be the case [54]. Perhaps the direct killing effects greatly make up for any anti-cancer immunity effects. In fact, there is evidence that radiation may enhance cancer immune response either by releasing tumor antigens that can be targeted or by destroying immunosuppressive cells such as immunosuppressive cells in the stroma [55,56].

In addition, we know that radiation can have inflammatory effects on normal structures such as rectal mucosa. In fact, the most common bothersome side effect with prostate radiation is loose bowels or diarrhea thought to be from local cell destruction and inflammation. The preceding considerations raise the question as to whether radiation would suppress immunity or enhance it.

Consistent with the published literature, in our experience, radiation led to a significant decrease in WBC and lymphocyte levels. The PSA always went down from pre-radiation levels (or remained stable), so the cancer was being killed or at least suppressed, while the CRP did not change. Maybe the pro-inflammatory effects and the anti-inflammatory effects balanced out. Or, as noted previously, CRP is just not that sensitive with lower volume cancer. It has been reported that CRP goes up with radiation treatments and is significantly higher at the end [8]. We did not see that.

Finally, with follow-up, the inflammatory cells start to recover, while the cancer cells continue to die (as reflected by a continually decreasing PSA). Any local acute effects in the normal tissues are usually resolved, though it is possible there is ongoing cell die off that can release pro- or anti-inflammatory mediators. Indeed, in our patients, lymphocytes and WBCs had significantly recovered from the end of treatment while the PSA continued to decrease. CRP, again, did not change significantly either way.

In our analysis, CRP remained spectacularly stable without significantly changing in our cohort at any time point.

## Conclusions

Our results demonstrate a lack of clinical utility for CRP as a marker of the inflammatory effects of non-metastatic prostate cancer. It has no correlation with PSA levels or trends before or after radiation therapy and with or without androgen ablation. It does not change with significant PSA decrease after androgen ablation and corresponding decrease in cancer activity. It does not change at the end of radiation therapy despite significant changes in lymphocytes and WBCs. It does not change with a three-month follow-up to correspond with either significant recovery of lymphocytes and WBCs or a decrease of radiation-induced inflammation.

CRP levels do not correspond with the immune effects of locally advanced prostate cancer or its treatment. Perhaps it will still have some long-term prognostic utility or in the metastatic setting, but it appears that for prostate cancer, resources would be put to better use searching for more sensitive inflammatory markers. Our results also bring in to question the role of CRP in inflammation induced by other malignancies.

## References

[1] Alberdi-Saugstrup M, Zak M, Nielsen S (2017). High-sensitive CRP as a predictive marker of long-term outcome in juvenile idiopathic arthritis. Rheumatol Int.

[2] Gewurz H, Mold C, Siegel J, Fiedel B (1982). C-reactive protein and the acute phase response. Adv Intern Med.

[3] Allin KH, Bojesen SE, Nordestgaard BG (2016). Inflammatory biomarkers and risk of cancer in 84,000 individuals from the general population. Int J Cancer.

[4] Solomon DH, Greenberg J, Curtis JR (2015). Derivation and internal validation of an expanded cardiovascular risk prediction score for rheumatoid arthritis: a Consortium of Rheumatology Researchers of North America Registry Study. Arthritis Rheumatol.

[5] Ong KL, Chung RW, Hui N (2020). Usefulness of certain protein biomarkers for prediction of coronary heart disease. Am J Cardiol.

[6] Heikkilä K, Ebrahim S, Lawlor DA (2007). A systematic review of the association between circulating concentrations of C reactive protein and cancer. J Epidemiol Community Health.

[7] Heikkilä K, Harris R, Lowe G (2009). Associations of circulating C-reactive protein and interleukin-6 with cancer risk: findings from two prospective cohorts and a meta-analysis. Cancer Causes Control.

[8] Koc M, Taysi S, Sezen O, Bakan N (2003). Levels of some acute-phase proteins in the serum of patients with cancer during radiotherapy. Biol Pharm Bull.

[9] Hall WA, Nickleach DC, Master VA (2013). The association between C-reactive protein (CRP) level and biochemical failure-free survival in patients after radiation therapy for nonmetastatic adenocarcinoma of the prostate. Cancer.

[10] Guo YZ, Pan L, Du CJ, Ren DQ, Xie XM (2013). Association between C-reactive protein and risk of cancer: a meta-analysis of prospective cohort studies. Asian Pac J Cancer Prev.

[11] Siemes C, Visser LE, Coebergh JW (2006). C-reactive protein levels, variation in the C-reactive protein gene, and cancer risk: the Rotterdam Study. J Clin Oncol.

[12] Gabay C, Kushner I (1999). Acute-phase proteins and other systemic responses to inflammation. N Engl J Med.

[13] Allin KH, Nordestgaard BG (2011). Elevated C-reactive protein in the diagnosis, prognosis, and cause of cancer. Crit Rev Clin Lab Sci.

[14] Saito K, Kihara K (2011). C-reactive protein as a biomarker for urological cancers. Nat Rev Urol.

[15] Thurner EM, Krenn-Pilko S, Langsenlehner U (2015). The elevated C-reactive protein level is associated with poor prognosis in prostate cancer patients treated with radiotherapy. Eur J Cancer.

[16] Van Hemelrijck M, Jungner I, Walldius G (2011). Risk of prostate cancer is not associated with levels of C-reactive protein and other commonly used markers of inflammation. Int J Cancer.

[17] Eklund CM, Tammela TL, Schleutker J, Hurme M (2009). C-reactive protein haplotype is associated with high PSA as a marker of metastatic prostate cancer but not with overall cancer risk. Br J Cancer.

[18] Markt SC, Rider JR, Penney KL (2014). Genetic variation across C-reactive protein and risk of prostate cancer. Prostate.

[19] Pierce BL, Biggs ML, DeCambre M (2009). C-reactive protein, interleukin-6, and prostate cancer risk in men aged 65 years and older. Cancer Causes Control.

[20] St Hill CA, Lutfiyya MN (2015). An epidemiological analysis of potential associations between C-reactive protein, inflammation, and prostate cancer in the male US population using the 2009-2010 National Health and Nutrition Examination Survey (NHANES) data. Front Chem.

[21] Stark JR, Li H, Kraft P (2009). Circulating prediagnostic interleukin-6 and C-reactive protein and prostate cancer incidence and mortality. Int J Cancer.

[22] Stikbakke E, Richardsen E, Knutsen T (2020). Inflammatory serum markers and risk and severity of prostate cancer: the PROCA-life study. Int J Cancer.

[23] Toriola AT, Laukkanen JA, Kurl S, Nyyssönen K, Ronkainen K, Kauhanen J (2013). Prediagnostic circulating markers of inflammation and risk of prostate cancer. Int J Cancer.

[24] Arthur R, Williams R, Garmo H (2018). Serum inflammatory markers in relation to prostate cancer severity and death in the Swedish AMORIS study. Int J Cancer.

[25] Elsberger B, Lankston L, McMillan DC, Underwood MA, Edwards J (2011). Presence of tumoural C-reactive protein correlates with progressive prostate cancer. Prostate Cancer Prostatic Dis.

[26] Gómez-Gómez E, Carrasco-Valiente J, Campos-Hernández JP (2019). Clinical association of metabolic syndrome, C-reactive protein and testosterone levels with clinically significant prostate cancer. J Cell Mol Med.

[27] Lehrer S, Diamond EJ, Mamkine B, Droller MJ, Stone NN, Stock RG (2005). C-reactive protein is significantly associated with prostate-specific antigen and metastatic disease in prostate cancer. BJU Int.

[28] Liao SG, Cheng HH, Lei Y (2016). C-reactive protein is a prognostic marker for patients with castration-resistant prostate cancer. Oncol Res Treat.

[29] Schnoeller TJ, Steinestel J, Steinestel K, Jentzmik F, Schrader AJ (2015). Do preoperative serum C-reactive protein levels predict the definitive pathological stage in patients with clinically localized prostate cancer?. Int Urol Nephrol.

[30] Zhou L, Cai X, Liu Q, Jian ZY, Li H, Wang KJ (2015). Prognostic role of C-reactive protein in urological cancers: a meta-analysis. Sci Rep.

[31] Liu ZQ, Chu L, Fang JM, Zhang X, Zhao HX, Chen YJ, Xu Q (2014). Prognostic role of C-reactive protein in prostate cancer: a systematic review and meta-analysis. Asian J Androl.

[32] Graff JN, Beer TM, Liu B, Sonpavde G, Taioli E (2015). Pooled analysis of C-reactive protein levels and mortality in prostate cancer patients. Clin Genitourin Cancer.

[33] Uchimoto T, Komura K, Fujiwara Y (2019). Prognostic impact of C-reactive protein-albumin ratio for the lethality in castration-resistant prostate cancer. Med Oncol.

[34] Xu L, Zhao Q, Huang S, Li S, Wang J, Li Q (2015). Serum C-reactive protein acted as a prognostic biomarker for overall survival in metastatic prostate cancer patients. Tumour Biol.

[35] Yamada Y, Sakamoto S, Rii J (2020). Prognostic value of an inflammatory index for patients with metastatic castration-resistant prostate cancer. Prostate.

[36] Beer TM, Lalani AS, Lee S (2008). C-reactive protein as a prognostic marker for men with androgen-independent prostate cancer: results from the ASCENT trial. Cancer.

[37] Ito M, Saito K, Yasuda Y (2011). Prognostic impact of C-reactive protein for determining overall survival of patients with castration-resistant prostate cancer treated with docetaxel. Urology.

[38] Nakashima J, Kikuchi E, Miyajima A, Nakagawa K, Oya M, Ohigashi T, Murai M (2008). Simple stratification of survival using bone scan and serum C-reactive protein in prostate cancer patients with metastases. Urol Int.

[39] Pond GR, Armstrong AJ, Wood BA, Leopold L, Galsky MD, Sonpavde G (2012). Ability of C-reactive protein to complement multiple prognostic classifiers in men with metastatic castration resistant prostate cancer receiving docetaxel-based chemotherapy. BJU Int.

[40] Prins RC, Rademacher BL, Mongoue-Tchokote S, Alumkal JJ, Graff JN, Eilers KM, Beer TM (2012). C-reactive protein as an adverse prognostic marker for men with castration-resistant prostate cancer (CRPC): confirmatory results. Urol Oncol.

[41] Rocha P, Morgan CJ, Templeton AJ, Pond GR, Naik G, Sonpavde G (2014). Prognostic impact of C-reactive protein in metastatic prostate cancer: a systematic review and meta-analysis. Oncol Res Treat.

[42] Lee S, Choe JW, Kim HK, Sung J (2011). High-sensitivity C-reactive protein and cancer. J Epidemiol.

[43] Shrotriya S, Walsh D, Bennani-Baiti N, Thomas S, Lorton C (2015). C-reactive protein is an important biomarker for prognosis tumor recurrence and treatment response in adult solid tumors: a systematic review. PLoS One.

[44] Lippi G, Montagnana M, Guidi GC (2009). Epidemiological association between C-reactive protein and prostate-specific antigen. Cancer.

[45] McArdle PA, Mir K, Almushatat AS, Wallace AM, Underwood MA, McMillan DC (2006). Systemic inflammatory response, prostate-specific antigen and survival in patients with metastatic prostate cancer. Urol Int.

[46] Turner N, Pestrin M, Galardi F, De Luca F, Malorni L, Di Leo A (2014). Can biomarker assessment on circulating tumor cells help direct therapy in metastatic breast cancer?. Cancers (Basel).

[47] Zandman-Goddard G, Peeva E, Shoenfeld Y (2007). Gender and autoimmunity. Autoimmun Rev.

[48] Morse MD, McNeel DG (2010). Prostate cancer patients on androgen deprivation therapy develop persistent changes in adaptive immune responses. Hum Immunol.

[49] Salman H, Bergman M, Blumberger N, Djaldetti M, Bessler H (2014). Do androgen deprivation drugs affect the immune cross-talk between mononuclear and prostate cancer cells?. Biomed Pharmacother.

[50] Kaczmarek P, Pokoca L, Niemirowicz J, Majewska E, Baj Z (2008). Effect of luteinizing hormone-releasing hormone (LHRH) analogue treatment on a cytokine profile in prostate cancer patients. Pharmacol Rep.

[51] Saylor PJ, Kozak KR, Smith MR (2012). Changes in biomarkers of inflammation and angiogenesis during androgen deprivation therapy for prostate cancer. Oncologist.

[52] Wu CT, Chen WC, Lin PY, Liao SK, Chen MF (2009). Androgen deprivation modulates the inflammatory response induced by irradiation. BMC Cancer.

[53] McMillan DC, Canna K, McArdle CS (2003). Systemic inflammatory response predicts survival following curative resection of colorectal cancer. Br J Surg.

[54] Rotstein S, Blomgren H, Baral E (1987). Incidence of infectious symptoms after radiation therapy for breast cancer: long-term effects. Acta Oncol.

[55] Polyak K (2006). The p27Kip1 tumor suppressor gene: still a suspect or proven guilty?. Cancer Cell.

[56] Orimo A, Gupta PB, Sgroi DC (2005). Stromal fibroblasts present in invasive human breast carcinomas promote tumor growth and angiogenesis through elevated SDF-1/CXCL12 secretion. Cell.

